# Negotiated control between the manual and visual systems for visually guided hand reaching movements

**DOI:** 10.1186/1743-0003-11-102

**Published:** 2014-06-12

**Authors:** K Han Kim, R Brent Gillespie, Bernard J Martin

**Affiliations:** 1 Washington State Department of Labor and Industries, Safety and Health Assessment and Research for Prevention Program, PO Box 44330, Olympia, WA 98504-4330, USA; 2 Department of Mechanical Engineering, The University of Michigan, Address: 2350 Hayward, Ann Arbor, MI 48109-2125, USA; 3 Department of Industrial and Operations Engineering, The University of Michigan, Address: 1205 Beal Avenue, Ann Arbor, MI 48109-2117, USA

**Keywords:** Coordination, Sensorimotor systems, Human motion simulation

## Abstract

**Background:**

Control of reaching movements for manual work, vehicle operation, or interactions with manual interfaces requires concurrent gaze control for visual guidance of the hand. We hypothesize that reaching movements are based on negotiated strategies to resolve possible conflicting demands placed on body segments shared by the visual (gaze) and manual (hand) control systems. Further, we hypothesize that a multiplicity of possible spatial configurations (redundancy) in a movement system enables a resolution of conflicting demands that does not require sacrificing the goals of the two systems.

**Methods:**

The simultaneous control of manual reach and gaze during seated reaching movements was simulated by solving an inverse kinematics model wherein joint trajectories were estimated from a set of recorded hand and head movements. A secondary objective function, termed *negotiation function*, was introduced to describe a means for the manual reach and gaze directing systems to balance independent goals against (possibly competing) demands for shared resources, namely the torso movement. For both systems, the trade-off may be resolved without sacrificing goal achievement by taking advantage of redundant degrees of freedom. Estimated joint trajectories were then compared to joint movement recordings from ten participants. Joint angles were predicted with and without the negotiation function in place, and model accuracy was determined using the root-mean-square errors (RMSEs) and differences between estimated and recorded joint angles.

**Results:**

The prediction accuracy was generally improved when negotiation was included: the negotiated control reduced RMSE by 16% and 30% on average when compared to the systems with only manual or visual control, respectively. Furthermore, the RMSE in the negotiated control system tended to improve with torso movement amplitude.

**Conclusions:**

The proposed model describes how multiple systems cooperate to perform goal-directed human movements when those movements draw upon shared resources. Allocation of shared resources can be undertaken by a negotiation process that is aware of redundancies and the existence of multiple solutions within the individual systems.

## Background

Many daily activities include hand reaching movements devised to bring the hand to a desired target. For accurate hand reaches, it is crucial to capture visual images of the environment and self (body parts). Visual information provides feedback for accurate guidance of movements [1,2] and plays an important role in the generation of movement plans as well as the calibration of movement systems with respect to the environment [3-5].

These perspectives suggest that reaching movements involve the coordination of two effector systems: the hand being guided to the target and the gaze being directed at the target. Simultaneously achieving accurate reaching and appropriately directing gaze is not a trivial task, as the central nervous system (CNS) has to control both the manual and visual systems, each of which relies on different sets of body segments and end-effectors. The CNS must solve complex problems from multiple layers of reference frame conversions [6], spatial transformation [7] and sensory-motor integration through feedback and feed-forward control [8].

Furthermore, the actions of these systems are not completely independent from one another, as they share common body segments (links). Both the arms/hands and neck/head are connected to the torso, thus the actions of one system often impose disturbances on the other. That is, hand movements may be accompanied by torso movements, which would also influence head and eye positions. The torso is in effect a resource shared by the manual and visual systems, and demands made on this shared resource may compete.

Hence in planning and executing visually guided hand reaching movements, the allocation of shared resources becomes critical to the manual system (MS) and visual system (VS). When demands on shared resources are in conflict, a rule must be established in a manner that allows these systems to compromise their demands on the shared resources. Thus it is hypothesized that the CNS employs a negotiation system (NS), which considers the demands from the MS and VS and allocates the contribution of the torso in a manner that serves the goals of both the MS and VS.

The negotiation between multiple systems is necessary because of the existence of shared resources (torso movement) and negotiation with resolution of conflict (benefiting both the MS and VS) is made possible by the existence of redundant degrees of freedom in both the MS and VS. Redundancy implies the existence of multiple spatial configurations of each linkage system that achieve the same hand reach position (or gaze direction in the case of the VS). By taking advantage of redundancy, conflicting demands on torso movement can be resolved, while satisfying the desired end effector movements. For example, the MS may require torso flexion while the VS requires torso extension. In such cases, both systems will have to surrender a portion of torso movement, but by virtue of redundancy, they may increase the utilization of the uniquely dedicated remaining body segments so that the aims of hand and gaze are both best fulfilled.

Given the complex multi-link system of the human body, the problem to solve is the determination of joint angle sets that closely mimic real movement realizations. When the available degrees of freedom (the number of joints in the body) is greater than the dimension of the task space (the number of coordinates required to describe the end-effector configuration), the solution space may be very large and the relationships between joint space variables and task space variables can be highly nonlinear. In robotics, a commonly used method for solving the inverse kinematics problem (determining joint angles given end-effector coordinates) is known as differential inverse kinematics. Differential inverse kinematics relies on inversion of the relationship between joint angular velocities and end-effector velocities, or inversion of kinematics in the velocity domain. The differentiated kinematics is linear in the joint and end-effector velocities, simplifying the process of inversion (solving for the joint velocities in terms of the end-effector velocities) (see [9] for a review). In the case of redundancy, where more than one feasible solution is possible, optimization is commonly used to identify the best solution given a pre-defined objective function. Such objective functions can be defined to minimize the (possibly weighted) sum of joint velocities [10-12], distance to mechanical joint limits [13], or distance to obstacles [14]. For our goal of modeling the coordination of two systems (the MS and VS) with shared body segments, however, an additional objective function is needed to implement negotiation.

Thus the primary goal of the framework developed here is to estimate the joint angles that place the end-effectors of the MS and VS in the desired locations. The secondary goal is to allocate the use of shared resources (shared body segments or links) for both systems according to a negotiation scheme. To validate this framework, human movements in three-dimensional seated right-hand reaching tasks were simulated with and without the negotiation scheme. These simulations took the form of solutions of the inverse kinematics of the redundant systems, as driven by recorded hand and head movements only. The benefit of the negotiation framework was then quantified by comparing simulated and recorded movements, in terms of the movements of all body segments. It is hypothesized that negotiation is a mechanism adopted by the CNS to control multi-segmental movements of the human body and therefore provides better predictions of body movements. This framework would also help to understand the interactions between the manual and visual systems in goal-directed movements.

## Methods

### Movement modeling

Seven revolute joints corresponding to the hand, forearm, upper arm and torso comprise the manual system variables, which can be represented by an angle vector **θ**_*m*_ = [*θ*_*m*1_,  *θ*_*m*2_,  *θ*_*m*3_,  *θ*_*m*4_,  *θ*_*m*5_,  *θ*_*m*6_,  *θ*_*m*7_]^T^. All joint variables were represented in the Denavit-Hartenberg convention [15,16], as listed in Table 1. Similarly, the visual system configuration **θ**_*v*_ = [*θ*_*v*1_,  *θ*_*v*2_,  *θ*_*v*3_,  *θ*_*v*4_,  *θ*_*v*5_,  *θ*_*v*6_]^T^ is described using six revolute joints corresponding to the head, neck, and torso. Since three joints of the torso (corresponding to flexion/extension, axial rotation, and lateral bending at the L5/S1 joint) are shared by both systems, the corresponding variables can be represented by common terms *θ*_1_, *θ*_2_, and *θ*_3_ and the above definitions can be rewritten as **θ**_*m*_ = [*θ*_1_,  *θ*_2_,  *θ*_3_,  *θ*_*m*4_,  *θ*_*m*5_,  *θ*_*m*6_,  *θ*_*m*7_]^T^ and **θ**_*v*_ = [*θ*_1_,  *θ*_2_,  *θ*_3_,  *θ*_*v*4_, *θ*_*v*5_,  *θ*_*v*6_]^T^, respectively. Also by conjoining **θ**_*m*_ and **θ**_*v*_, **θ** = [*θ*_1_,  *θ*_2_,  *θ*_3_,  *θ*_*m*4_,  *θ*_*m*5_,  *θ*_*m*6_,  *θ*_*m*7_,  *θ*_*v*4_,  *θ*_*v*5_,  *θ*_*v*6_]^T^. With the MS, upper arm axial rotation (*θ*_*m*6_) is equivalent to internal/external rotation at the shoulder. Wrist joints (3 additional degrees of freedom: flexion/extension, pronation/supination, and ulnar/radial deviations) were not considered, to similarly match the VS joint count.

**Table 1:** Link segment compositions for the manual and visual system

**Manual system**			**Visual system**	
**Variable**	**Joint movement**	**Positive rotation**	**Variable**	**Joint movement**
*θ*_*m*1_	Torso flexion/extension	Extension	*θ*_*v*1_	Torso flexion/extension
*θ*_*m*2_	Torso axial rotation	Counter-clockwise	*θ*_*v*2_	Torso axial rotation
*θ*_*m*3_	Torso lateral bending	Leftward	*θ*_*v*3_	Torso lateral bending
*θ*_*m*4_	Shoulder flexion/extension	Flexion	*θ*_*v*4_	Neck/Head flexion/extension
*θ*_*m*5_	Shoulder abduction/adduction	Abduction	*θ*_*v*5_	Neck/Head axial rotation
*θ*_*m*6_	Upper arm axial rotation	Counter-clockwise	*θ*_*v*6_	Neck/Head lateral bending
*θ*_*m*7_	Elbow flexion/extension	Flexion		

The position of the right hand **p**_m_, which is the manual system end-effector, is described by Cartesian coordinates as in $p_{m}={\left[p_{\text{hand}\;\left(x\right)}^{\text{global}},\;p_{\text{hand}\;\left(y\right)}^{\text{global}},p_{\text{hand}\;\left(z\right)}^{\text{global}}\right]}^{T}=f_{m}\left(\theta_{m}\right)$ where $p_{\text{hand}\;\left(x\right)}^{\text{global}},$$p_{\text{hand}\;\left(y\right)}^{\text{global}}$ and $p_{\text{hand}\;\left(z\right)}^{\text{global}}$ denote *x*- (positive rightward), *y*- (positive forward), and *z*- (positive upward) coordinates of the hand in a global coordinate system, with the origin coinciding with the L5/S1 joint position. The symbol *f*_m_ denotes a function of the direct (forward) kinematics of hand reaching movements describing hand position as a function of joint angles.

The end-effector position of the visual system is defined as $p_{v}={\left[p_{\text{target}\;\left(x\right)}^{\text{head}},\;p_{\text{target}\;\left(y\right)}^{\text{head}},\;p_{\text{target}\;\left(z\right)}^{\text{head}}\right]}^{T}=f_{v}\left(\theta_{v}\right),$ in which $p_{\text{target}\;\left(x\right)}^{\text{head}}$, $p_{\text{target}\;\left(y\right)}^{\text{head}}$ and $p_{\text{target}\;\left(z\right)}^{\text{head}}$ represent the position of the target along the *x*-, *y*-, and *z*-axes in a head-centered reference frame. The symbol *f*_*v*_ denotes the direct kinematics function for the visual system.

The goal of the present model is to estimate a joint angle vector (**θ**) based on the recorded end-effector position using the inverse kinematics function $f_{m}^{-1}\left(p_{m}\right)$, or $f_{v}^{-1}\left(p_{v}\right)$. Using the differential kinematics method, the velocity $\dot{p}$ of the end-effector position **p** can be obtained using

(1) $$\dot{p}=\frac{dp}{\text{dt}}=\frac{dp}{d\theta }\;\frac{d\theta }{\text{dt}}=\frac{\partial f\left(\theta \right)}{\partial \theta }\dot{\theta }=J\left(\theta \right)\dot{\theta }$$

where **J** is a Jacobian matrix that is a function of the given joint configuration **θ**. Equation 1 can be adapted to represent either the manual system ${\dot{p}}_{m}=J_{m}\dot{\theta }$ or visual system ${\dot{p}}_{v}=J_{v}\dot{\theta }$, where **J**_*m*_ and **J**_*v*_ represent the Jacobian matrices for the manual and visual system, respectively. The solution of inverse kinematics can then be obtained by solving for the joint angular velocities ${\dot{\theta }}_{m}$ and ${\dot{\theta }}_{v}$ and integrating with feedback stabilization [9,17].

Due to the existence of redundant degrees of freedom (non-square matrix **J**), the inverse of **J** does not exist. However, a weighted pseudo-inverse (denoted **J**^†^) may be used in which the squared sum of all joint velocities is minimized [10,11]. Thus from Equation 1,

(2) $$\dot{\theta }=W^{-1}J^{T}{\left(JW^{-1}J^{T}\right)}^{-1}\dot{p}=J^{†}\dot{p}$$

where **W** is a weighting matrix that characterizes the instantaneous contribution of each joint. In this study, **W** is a diagonal matrix whose entries are the peak magnitudes of joint velocities in recorded individual movements.

A secondary objective function implements the negotiation scheme (*negotiation function*). This function reconfigures the joint angles of the link system without changing the end-effector position by utilizing the redundant degrees of freedom. The projection matrix $\left(I-J^{†}J\right)\;{\dot{\theta }}_{0}$, where **I** represents the identity matrix, can be used to project an arbitrary vector ${\dot{\theta }}_{0}$ into the null space of **J**. Thus from Equation 2, the manual system equation can be adapted as follows.

(3) $$\dot{\theta }=J_{m}^{†}{\dot{p}}_{m}+\left(I-J_{m}^{†}J_{m}\right){\dot{\theta }}_{0}$$

As described in [14,17], multiplying Equation 3 by **J**_*v*_ would result in $J_{v}\dot{\theta }=J_{v}J_{m}^{†}{\dot{p}}_{m}+J_{v}\left(I-J_{m}^{†}J_{v}\right){\dot{\theta }}_{0}$. Solving for ${\dot{\theta }}_{0}$ would thus result in ${\dot{\theta }}_{0}={\left[J_{v}\left(I-J_{m}^{†}J_{m}\right)\right]}^{†}\left(J_{v}\dot{\theta }-J_{v}J_{m}^{†}{\dot{p}}_{m}\right)$. By substituting for ${\dot{\theta }}_{0}$ from Equation 3:

(4) $$\dot{\theta }=J_{m}^{†}{\dot{p}}_{m}+\left(I-J_{m}^{†}J_{m}\right){\left[J_{v}\left(I-J_{m}^{†}J_{m}\right)\right]}^{†}\left(J_{v}\dot{\theta }-J_{v}J_{m}^{†}{\dot{p}}_{m}\right)$$

Since $\left(I-J_{m}^{†}J_{m}\right)$ is Hermitian and idempotent, Equation 4 can be simplified as [14]:

(5) $$\dot{\theta }=J_{m}^{†}{\dot{p}}_{m}+{\left[J_{v}\left(I-J_{m}^{†}J_{m}\right)\right]}^{†}\left({\dot{p}}_{v}-J_{v}J_{m}^{†}{\dot{p}}_{m}\right)$$

Then **θ** can be obtained from the numerical integration of $\dot{\theta }$ and the cumulative error of the end-effector position predictions is reduced by a feedback control algorithm [14].

In this study, recorded movements (described below) provided end-effector velocity ${\dot{p}}_{m}$ and ${\dot{p}}_{v},$ and initial posture angles used for input parameters. Since the ultimate goal of this model is to compare torso movements predicted by the MS, VS, and NS respectively, the torso joint variables were included as a part of each system and each system was designed to generate joint movement estimations as follow: First, the manual and visual systems were separately considered to compute the corresponding joint angle estimations ( ${\hat{\theta }}_{m}$ and ${\hat{\theta }}_{v},$ respectively) using Equation 3, with θ_0_ set to a null vector. Subsequently Equation 5 was employed to construct the negotiation system and compute the corresponding joint angle estimations $\left({\hat{\theta }}_{n}\right)$.

### Movement recording

#### Participants

Five males and five females with a mean age of 22.3 ± 1.8 years (mean ± SD) participated in movement recording as paid volunteers. All participants were right-handed, free from any known musculoskeletal or neurological disorders, and had normal vision (20/20 or better) without corrective lenses. Mean stature and body weight were 170.9 cm (SD: ± 12.0) and 67.2 kg (SD: ± 16.3), respectively.

#### Equipment

**Figure 1:**
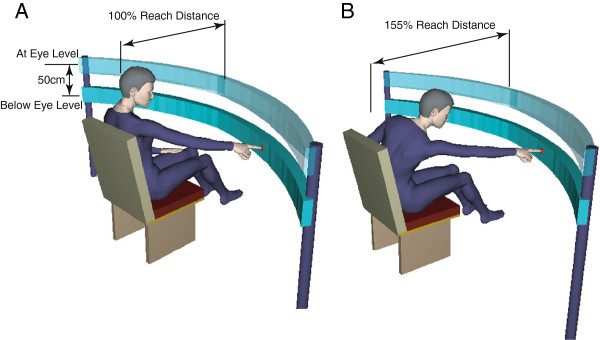
**Target configurations. A**: 100% reach distance. **B**: 155% reach distance.

Visual targets were placed on an arc (radius = 115 cm, arc length = 300 cm) set horizontally in front of the participant (Figure 1). The arc position was adjusted so that the mid-point of the arc coincided with the mid-sagittal plane of each participant. The elevation of the arc was set either at eye level or 50 cm below eye level (denoted as at-eye and below-eye level, respectively). The horizontal forward distance between the arc and the participant’s sternum was either 100% (Figure 1A) or 155% (Figure 1B) of the individual hand reach distance. Reach distance was defined as the length between the right acromion process and the tip of the right index finger while the upper arm, forearm, and hand were extended horizontally at shoulder level. The mean reach distance across all participants was 65 cm.

Each target was composed of alphanumeric characters (0 – 9, A, C, E, F, H, L, U, or P) displayed on a seven-segment LED whose visual angle was approximately 0.5°. Four targets were placed in each of the left and right hemispheres. In the 100% reach distance condition, the interval between the targets was approximately 15°, and the leftmost and rightmost target positions were approximately ± 60° of azimuth with respect to the mid-sagittal plane, respectively. In the 155% reach distance condition, the target interval was approximately 10° and the most eccentric positions corresponded to ± 40°. A separate LED display, used as the initial fixation point, was placed in the mid-sagittal plane at eye level for all arc elevation settings.

The participant was seated on a chair (seat pan height = 40 cm, seat pan width = 50 cm, back support height = 58 cm). A pad equipped with a micro switch was placed on the participant’s right lap and served as the home position of the right index finger. The position of the lap switch was adjusted so that the elbow-included angle was approximately 90° when the index finger was placed on the button. The entire room was dimly lit during the experiment.

#### Movement recording

An electromagnetic motion capture system (Flock of Birds™, Ascension Technology) with five sensors placed on the forehead, upper torso (C7), lower torso (L5), upper arm, and right hand, was used to record the movements of the head, neck, clavicle, torso, upper arm, forearm and hand. A splint was fastened with an elastic band to the palmar side of the index finger to maintain its posture constant throughout the experiments so that the coordinates of the six degree of freedom sensor on the right hand could be used to estimate the fingertip position while imposing minimal interference on the reach task. The Cartesian coordinates and orientations of the sensors were used to estimate the joint center locations [18,19]: for example, the sensor placed on the upper arm provides the position of the shoulder and elbow using the measured upper arm length. Similarly, clavicle movements were estimated from the sensor at the upper torso and computed position of the shoulder joint. The sensor movements were sampled at 25 Hz, and trajectories of each landmark were smoothed off-line by a second order Butterworth low-pass filter with a 6 Hz cutoff frequency. Since the electromagnetic motion capture system is sensitive to electromagnetic interference, testing equipment including the target arc and seat were made of non-metallic materials such as wood and composites. Linearity within the volume of measure was verified during the calibration procedure.

#### Procedures

When the initial fixation point was illuminated, accompanied by a 500 Hz signal tone with a 0.1 s duration, the participant was asked to align the nasion with the initial fixation point and depress the switch on the right lap pad with the right index finger. This defined the resting initial posture. After a delay of 2 seconds, the initial fixation display was turned off and a target was displayed at a randomly selected eccentricity. A 2000 Hz tone with a 0.1 s duration signaled the participant to initiate the reach movement. The participant was asked to point just below the target with the right index finger, which activated the micro switch placed on the fingertip to signal the completion of the hand movement. The alphanumeric characters displayed on the active target were changed once per second. The participant was asked to read each alphanumeric character aloud throughout the trials to ensure that gaze was maintained on target. The target was turned off 2 seconds after fingertip contact.

Each block of target presentations was composed of sixteen trials (8 target locations × 2 replications) for a given target arc distance and elevation. A total of four blocks (2 distances × 2 arc elevations) were recorded for each participant. A three-minute break was provided at the completion of each block. The order of block presentation was balanced and randomized across participants.

The procedures were reviewed and approved by the University of Michigan Health Sciences Institutional Review Board, and all participants signed an informed consent prior to the experiment.

#### Data processing

Movement onset was determined by the start of either hand or head movement, whichever occurred first. The hand movement onset was determined by the activation of the microswitch. The head movement onset was estimated by off-line analysis. Specifically, the start of a head movement was defined as the time when the head had been stationary for the previous 120 ms (3 consecutive sampling frames) and engaged in active rotation for the next 120 ms. The threshold for active head rotation was defined as an angular velocity ≥ 25°/s. The completion of movement was determined by the fingertip microswitch contact with a target zone.

Body segment angles were calculated from the joint center positions estimated from sensor positions [18,19]. For the torso, for example, the vectors from the L5/S1 to the C7/T1 and right shoulder joint were used to construct a rotation matrix describing torso orientation, from which the associated three joint angles (*θ*_1_: flexion/extension, *θ*_2_: axial rotation, and *θ*_3_: lateral bending) were calculated as

$$R_{\text{torso}}^{\text{global}}=\left[\begin{array}{ccc} \cos\theta_{1}\cos\theta_{3}+\sin\theta_{1}\sin\theta_{2}\sin\theta_{3} & -\sin\theta_{1}\cos\theta_{2} & \cos\theta_{1}\sin\theta_{3}-\cos\theta_{3}\sin\theta_{1}\sin\theta_{2}\\ \cos\theta_{3}\sin\theta_{1}-\cos\theta_{1}\sin\theta_{2}\sin\theta_{3} & \cos\theta_{1}\cos\theta_{2} & \sin\theta_{1}\sin\theta_{3}+\cos\theta_{1}\cos\theta_{3}\sin\theta_{2}\\ -\cos\theta_{2}\sin\theta_{3} & -\sin\theta_{2} & \cos\theta_{2}\cos\theta_{3} \end{array}\right]$$

In a similar way, the shoulder angles were calculated from the shoulder-to-elbow and hand-to-elbow vectors, and neck/head angles from the nasion-to-C7T1 and right-to-left tragion vectors. All joint angles were defined as listed in Table 1.

### Model performance analyses

#### Model accuracy evaluation

The joint angles estimated by the MS, VS and NS were compared with recorded movements in order to evaluate the accuracy performance of each model. Specifically, the joint angle error at movement completion (end-posture errors) and root mean square error (RMSE) throughout the movement duration were computed with respect to the corresponding recorded joint movements. RMSE was computed by

$$\text{RMSE}=\sqrt{\frac{\sum_{t=1}^{n}{\left({\hat{\theta }}_{i}\left(t\right)-\theta_{i}\left(t\right)\right)}^{2}}{n},}$$

where ${\hat{\theta }}_{i}\left(t\right)$ and *θ*_*i*_(*t*) denote the estimated and recorded angle of the *i*-th joint at the *t*-th measurement sample, with a total number of measurement samples *n* for the given trial. Errors from the NS model were compared with the MS and VS model errors. All joints including the torso and upper extremity in the MS and the torso and neck/head in the VS (Table 1) were considered and compared with the corresponding joint in the NS, respectively. Statistical significance was tested using bootstrap confidence intervals, which measures the empirical distribution of the parameters of interest by re-sampling the original observations. Bootstraping is known to be useful when common parametric statistical analysis may not be employed due to errors not normally distributed [20-22].

#### Allocation of torso joint contribution

It was hypothesized that the NS weighs the demands for the torso joint use from the MS and VS, then allocates torso contribution for each system depending on the assessed weight. Thus if the prediction by the NS is closer to the prediction by the MS than the prediction by the VS, it was interpreted that the NS has allocated larger torso joint contribution to the MS than the VS, or vice versa. Specifically, the magnitudes of differences in torso joint angles were compared between the NS and MS estimations and between the NS and VS estimations, and their respective ratio to the sum of difference magnitudes was estimated using:

(6) $$A_{M_{i}}=1-\frac{\left|{\hat{\theta }}_{M_{i}}-{\hat{\theta }}_{N_{i}}\right|}{\left|{\hat{\theta }}_{M_{i}}-{\hat{\theta }}_{N_{i}}\right|\left|{\hat{\theta }}_{V_{i}}-{\hat{\theta }}_{N_{i}}\right|}$$

where *A*_*Mi*_ denotes the proportion of torso movement contribution allocated to the MS for the *i*-th joint angle, ${\hat{\theta }}_{M_{i}},{\hat{\theta }}_{V_{i}}\;\mathrm{and}\;{\hat{\theta }}_{N_{i}}$ denote the *i*-th joint angle at movement completion estimated by the MS, VS, and NS, respectively. From its definition, *A*_*Mi*_ approaches 1 as the NS estimation approaches MS estimation. Conversely, *A*_*Mi*_ approaches 0 as the NS estimation approaches the VS estimation. *A*_*Mi*_ was computed for three torso joint angles for each trial and averaged for different targets, grouped by distances (100 vs. 155% reach distance), elevations (at eye level vs. below eye level), and azimuths (two targets at the leftmost, middle, and rightmost positions). Data processing and modeling work were performed using Matlab 2009 (Mathworks, Natick, MA).

## Results

### Overall accuracy estimation

The RMSEs were overall improved when the NS was taken into account in the model, when compared to the models with the MS and VS alone (Table 2). Specifically, the NS showed smaller RMSEs than the MS (*p* < 0.05) for all torso joints (flexion/extension, axial rotation, and lateral bending) and elbow flexion/extension joint (*p* < 0.05). The RMSEs were also smaller for the shoulder joint angles but statistical significance was not reached. Similarly, when compared to the model with the VS, the NS model showed smaller RMSE (*p* < 0.05) for all neck joints (flexion/extension, axial rotation and lateral bending).

**Table 2:** RMSE in joint angle estimations

	**MS**			**VS**			**NS**			**NS error reduction from MS**
	**Mean**	**SE**	**95% CI**	**Mean**	**SE**	**95% CI**	**Mean**	**SE**	**95% CI**	
*θ*_1_	2.43	0.08	[2.27, 2.58]	1.88	0.08	[1.72, 2.03]	1.44	0.06	[1.33, 1.55]	*41%
*θ*_2_	5.49	0.15	[5.19, 5.79]	6.30	0.18	[5.94, 6.66]	4.13	0.11	[3.93, 4.34]	*25%
*θ*_3_	3.14	0.11	[2.92, 3.35]	3.43	0.13	[3.17, 3.70]	2.39	0.10	[2.20, 2.58]	*24%
*θ*_m4_	10.98	0.33	[10.33, 11.63]				10.39	0.32	[9.75, 11.02]	5%
*θ*_m5_	13.45	0.34	[12.77, 14.12]				13.12	0.35	[12.43, 13.80]	2%
*θ*_m6_	10.99	0.30	[10.40, 11.58]				10.92	0.31	[10.32, 11.52]	1%
*θ*_m7_	7.88	0.20	[7.50, 8.27]				7.04	0.17	[6.71, 7.37]	*11%
*θ*_v4_				1.43	0.06	[1.32, 1.54]	1.27	0.06	[1.16, 1.38]	
*θ*_v5_				6.25	0.22	[5.83, 6.68]	3.23	0.09	[3.06, 3.40]	
*θ*_v6_				6.21	0.16	[5.88, 6.53]	3.96	0.11	[3.74, 4.18]	

MS: Manual system. VS: Visual system. NS: Negotiation system. SE: Standard error. 95% CI: 95 percentile confidence interval. *indicates a statistical significance at *p* < 0.05.

The decreased RMSE for the NS was estimated by the ratios of (RMSE_MS_ –RMSE_NS_)/RMSE_MS_ and (RMSE_VS_ –RMSE_NS_)/RMSE_VS_, which indicated that the NS reduces RMSE by 16% and 30% on average when compared to the MS and VS, respectively. The improvements in RMSE are most prominent for torso flexion/extension (41%) when compared to the MS and neck axial rotation (48%) when compared to the VS, respectively.

Similar results were found for the error magnitudes at movement completion (Table 3). Errors for torso (flexion/extension, axial rotation, and lateral bending), shoulder (flexion/extension), and elbow (flexion/extension) joints were significantly smaller (*p* < 0.05) for the NS than the MS model. Similarly, The NS showed smaller errors than the VS (*p* < 0.05) for all torso and neck joints. On average, the error magnitudes are 15 and 39% less for the NS than the MS and VS, respectively. The largest error reduction by the NS was found in torso (35%) and neck (60%) axial rotations with respect to the MS and VS, respectively.

**Table 3:** End posture errors in joint angle estimations

	**MS**			**VS**			**NS**			**NS Error Reduction from MS**
	**Mean**	**SE**	**95% CI**	**Mean**	**SE**	**95% CI**	**Mean**	**SE**	**95% CI**	
*θ*_1_	2.69	0.1	[2.49, 2.88]	2.94	0.13	[2.68, 3.19]	2.01	0.09	[1.84, 2.19]	*25%
*θ*_2_	7.99	0.25	[7.49, 8.48]	8.65	0.27	[8.13, 9.17]	5.19	0.16	[4.88, 5.51]	*35%
*θ*_3_	4.48	0.18	[4.13, 4.83]	5.54	0.22	[5.12, 5.96]	3.48	0.16	[3.18, 3.79]	*22%
*θ*_m4_	14.72	0.48	[13.78, 15.66]				13.68	0.47	[12.76, 14.60]	*7%
*θ*_m5_	16.63	0.52	[15.61, 17.64]				16.67	0.55	[15.59, 17.74]	0%
*θ*_m6_	13.92	0.44	[13.07, 14.78]				13.34	0.44	[12.47, 14.21]	4%
*θ*_m7_	8.67	0.27	[8.14, 9.20]				7.80	0.25	[7.32, 8.28]	*10%
*θ*_v4_				1.80	0.08	[1.65, 1.95]	1.47	0.08	[1.32, 1.62]	
*θ*_v5_				9.17	0.33	[8.52, 9.82]	3.68	0.13	[3.42, 3.93]	
*θ*_v6_				8.97	0.26	[8.47, 9.47]	4.63	0.15	[4.34, 4.92]	

MS: Manual system. VS: Visual system. NS: Negotiation system. SE: Standard error. 95% CI: 95 percentile confidence interval. *indicates a statistical significance at *p* < 0.05.

### Torso movement profiles

**Figure 2:**
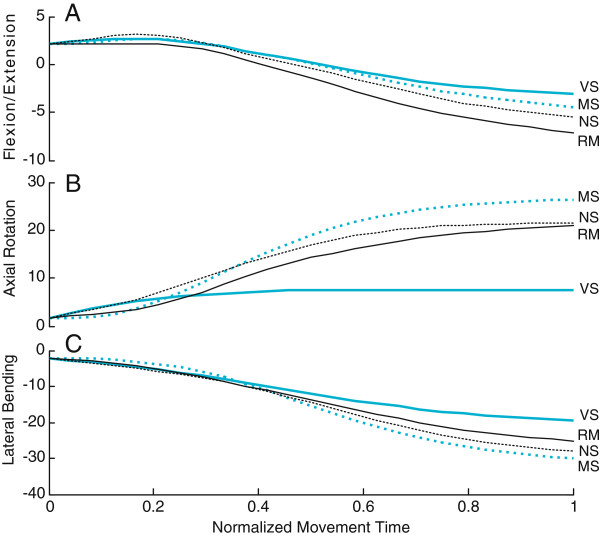
**Average torso angle profiles.** Angle profiles were averaged across all participants reaching for the leftmost target (60° mean target azimuth) at the 100% reach distance at eye level. **A**: flexion/extension. **B**: axial rotation. **C**: lateral bending. MS: Manual System Model. VS: Visual System Model. NS: Negotiation System Model. RM: Recorded Movements.

Since the distinctive role of the NS is to control the shared (torso) joints based on the MS and VS demands, torso movement profiles were investigated more in detail in this and the following sections. For all torso joints including flexion/extension, axial rotation and lateral bending, the model with the NS generally showed the closest estimation to the recorded movement when compared to the models with either the MS or VS alone. For example, in torso movement profiles averaged across all participants reaching for the leftmost target at the 100% reach distance at eye level, the flexion/extension angle in the recorded movement (RM) was -8°(flexion) at the end of the movement (Figure 2A). While the VS and MS estimated -3° and -4° flexion, the NS estimated a larger flexion (-6°), which is the closest to the recorded movement.In the same condition, the recorded movement showed leftward axial rotation increasing to 18° (Figure 2B). When model estimations were compared, all models (MS, VS, and NS) showed leftward axial rotations in agreement with the recorded movement. However, when compared to recorded movements, the MS shows an error “overshooting” up to 26°, while the VS showed an error “undershooting” to 8°. With the NS, the estimation was a slight overshoot to 21° which is in-between the MS and VS predictions. Accordingly, the NS provides estimation closest to the recorded movement. A similar tendency was observed from lateral bending angles (Figure 2C).

### Allocation of torso joint contribution

#### Torso flexion/extension joint

**Figure 3:**
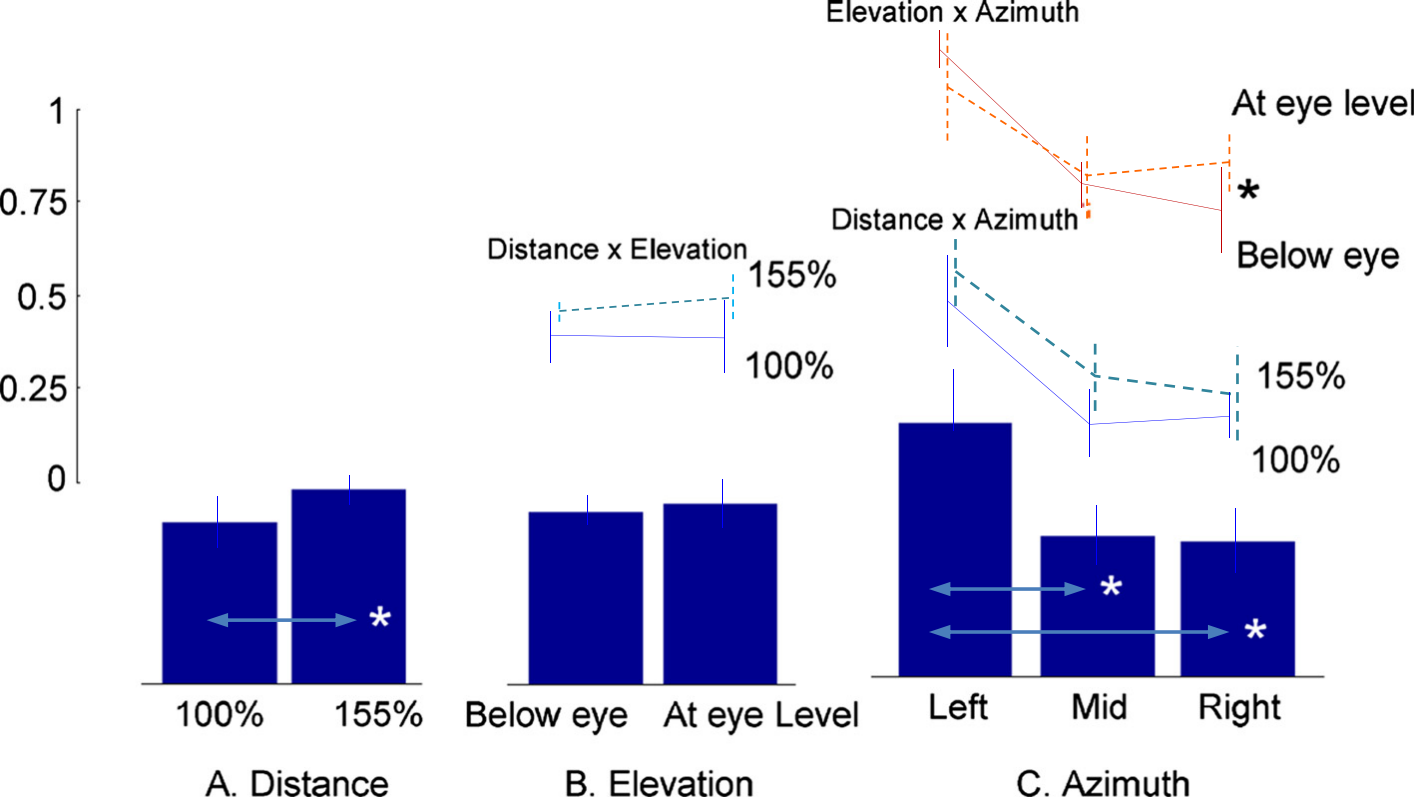
**Proportion of torso movement contribution allocated to the MS (torso flexion/extension).** Values on the *y*-axis are in a [0,1] scale and unitless (as defined in Equation 6). A value > 0.5 indicates that a larger torso movement contribution is allocated to the MS than the VS. *denotes a statistically significant difference at *p <* 0.05. **A**: Distance, **B**: Elevation, and **C**: Azimuth effects.

The torso joint contribution for each system allocated by the NS was estimated using Equation 6. The overall mean allocation ratio to the MS (all targets) is 0.47 (SE: ±0.02), which indicates that the allocations of torso contributions to the MS and VS are approximately similar. However, the MS allocation ratio was significantly larger (*p* < 0.05) for the 155% reach distance (0.52 ± 0.02) than the 100% reach distance (0.43 ± 0.03), as illustrated in Figure 3. Similarly, targets on the left side are associated with a larger (*p* < 0.05) allocation to the MS (0.68 ± 0.04) than those in the mid-sagittal plane (0.38 ± 0.04) or on the right side (0.36 ± 0.04), as shown in Figure 3. However, target elevation (either below or at eye level) does not significantly influence the allocation. Similarly, neither target distance × elevation nor target distance × azimuth interaction showed significant effects. However target elevation × azimuth shows a significant interaction effect (*p* < 0.05). Pairwise comparisons using Bonferroni corrections indicate that on the right side, the allocation ratio to the MS is greater for targets at eye level than targets below eye level (*p* < 0.05).

#### Torso axial rotation joint

**Figure 4:**
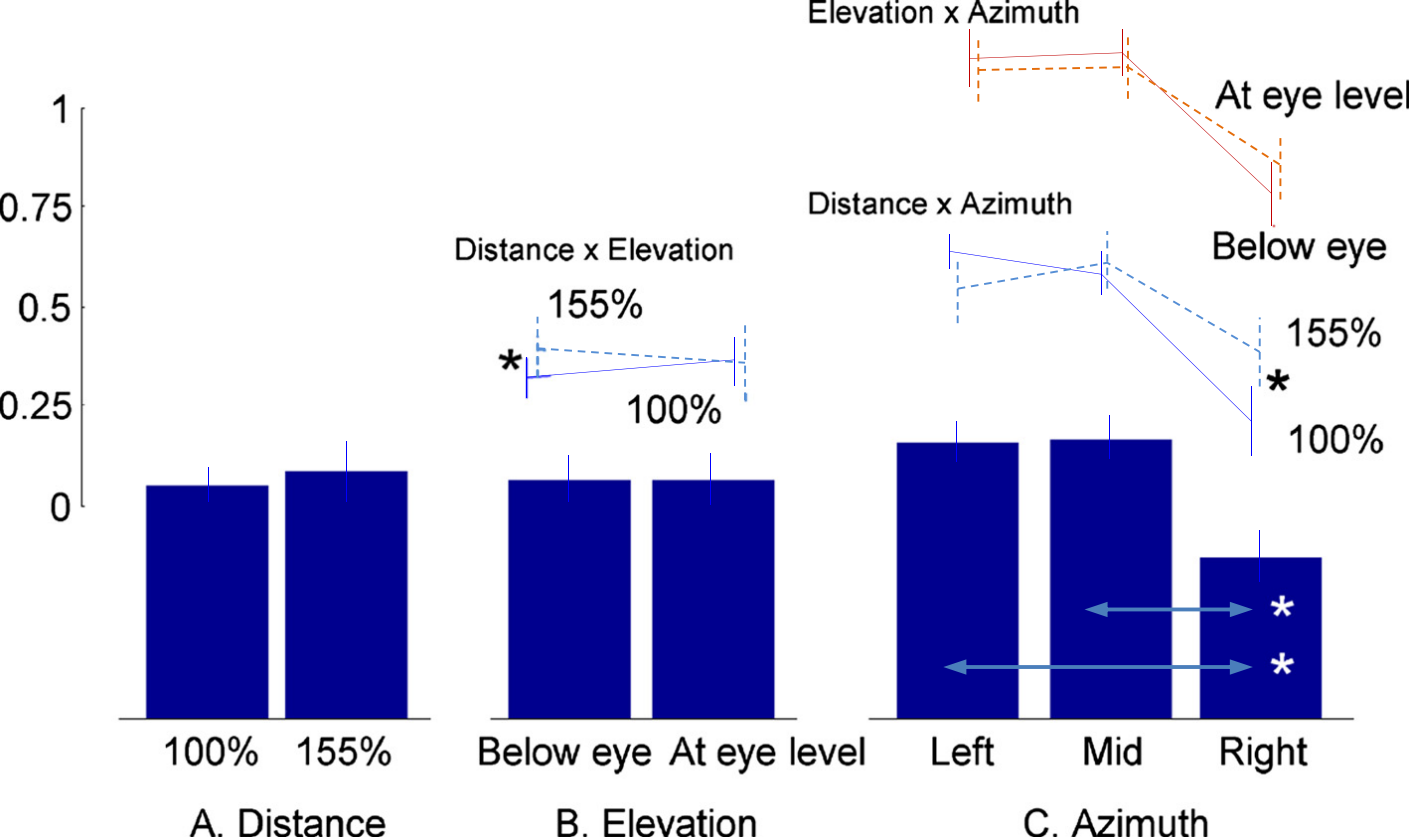
**Proportion of torso movement contribution allocated to the MS (torso axial rotation).** Values on the *y*-axis are in a [0,1] scale and unitless (as defined in Equation 6). A value > 0.5 indicates that a larger torso movement contribution is allocated to MS than VS. *denotes a statistically significant difference at *p <* 0.05. **A**: Distance, **B**: Elevation, and **C**: Azimuth effects.

The overall mean allocation ratio to the MS for axial rotation was 0.60 (SE 0.02) which indicates that a significantly larger (*p <* 0.05) allocation was made to the MS than the VS. Neither target distance nor elevation induces statistically significant effects (Figure 4). However, target azimuth was a significant factor (*p <* 0.05). The MS allocation ratio was smaller (*p <* 0.05) for targets on the right side (0.40 ± 0.03) than for targets in the mid-sagittal plane (0.70 ± 0.02) or on the left side (0.69 ± 0.02). A significant distance × elevation interaction effect (*p <* 0.05) indicates that allocation to the MS is larger for 155% than 100% reach distance for targets below eye level, while it is comparable for those at eye level. Similarly, larger allocations are made to the MS for targets at 155% than 100% reach distance on the right side, but they are comparable for targets on the left side and in the mid-sagittal plane (distance × azimuth interaction, *p <* 0.05). However, the elevation × azimuth interaction was not statistically significant.

#### Torso lateral bending joint

**Figure 5:**
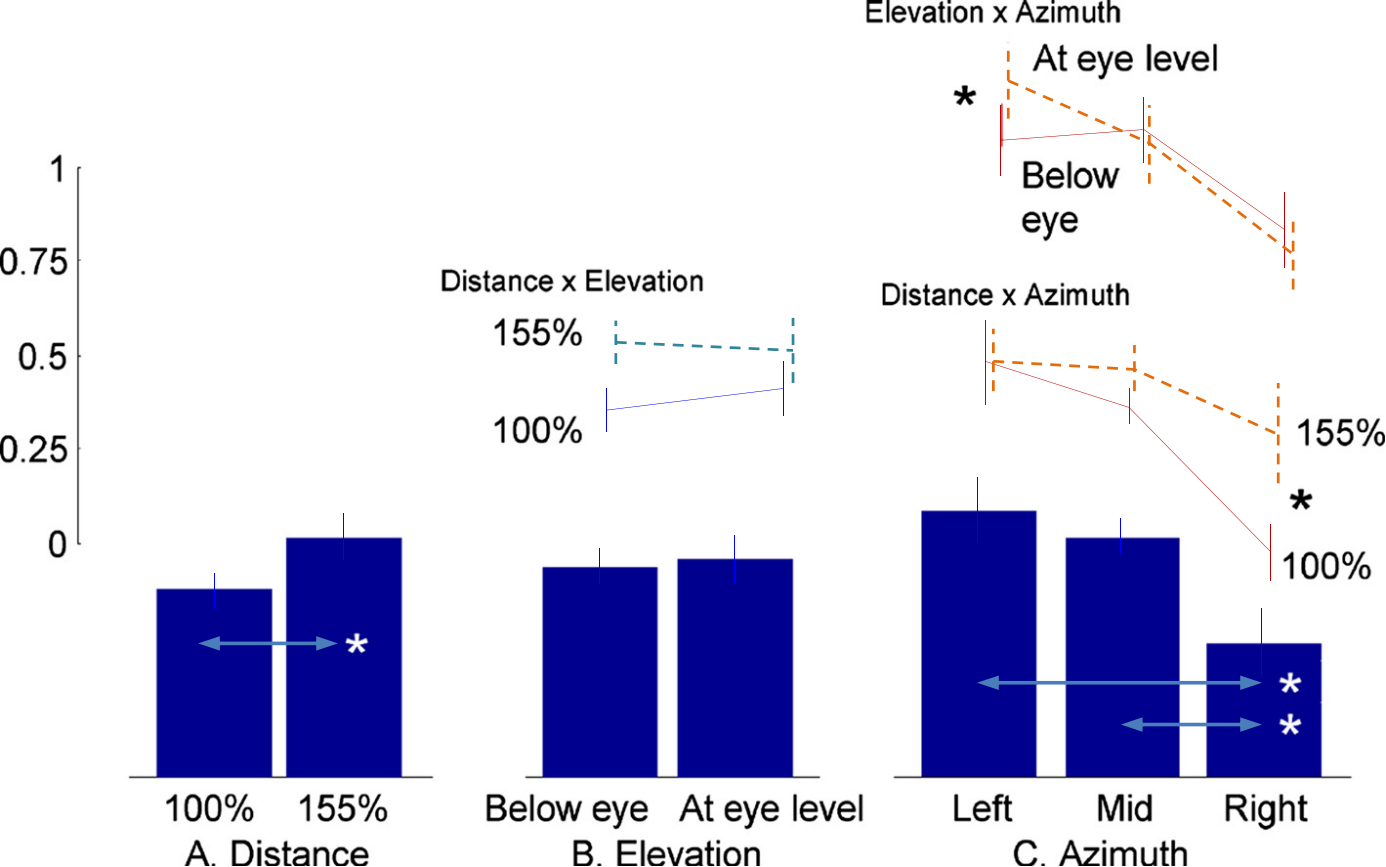
**Proportion of torso movement contribution allocated to MS (torso lateral bending).** Values on the *y*-axis are in a [0,1] scale and unitless (as defined in Equation 6). A value > 0.5 indicates that a larger torso movement contribution is allocated to MS than VS. *denotes a statistically significant difference at *p <* 0.05.** A**: Distance, **B**: Elevation, and **C**: Azimuth effects.

The overall mean allocation ratio to the MS for lateral bending was 0.57 ± 0.02, which indicates that a significantly larger (*p* < 0.05) allocation is made to the MS than the VS (Figure 5). Both reach distance and target azimuth induce statistically significant differences (*p <* 0.05). Specifically, the 155% reach distance induces a larger (0.64 ± 0.03) MS allocation ratio than the 100% reach distance (0.50 ± 0.02). For different target azimuths, pairwise comparisons indicate that MS allocations are smaller for targets on the right side (0.40 ± 0.04) than targets in the mid-sagittal plane (0.67 ± 0.02) and on the left side (0.71 ± 0.04). The distance × azimuth interaction is also significant (*p* < 0.05), as MS allocations are smaller for the 100% than 155% reach distance, and the corresponding difference becomes more predominant for targets on the right side than in the mid-sagittal plane or on the left side (*p <* 0.05). Also elevation × azimuth interaction indicates that targets below eye level are associated with a smaller MS allocation ratio than targets at eye level on the left side (*p <* 0.05), while differences are diminished for mid-sagittal plane and right side targets.

### Torso movement amplitude and RMSE improvements

**Figure 6:**
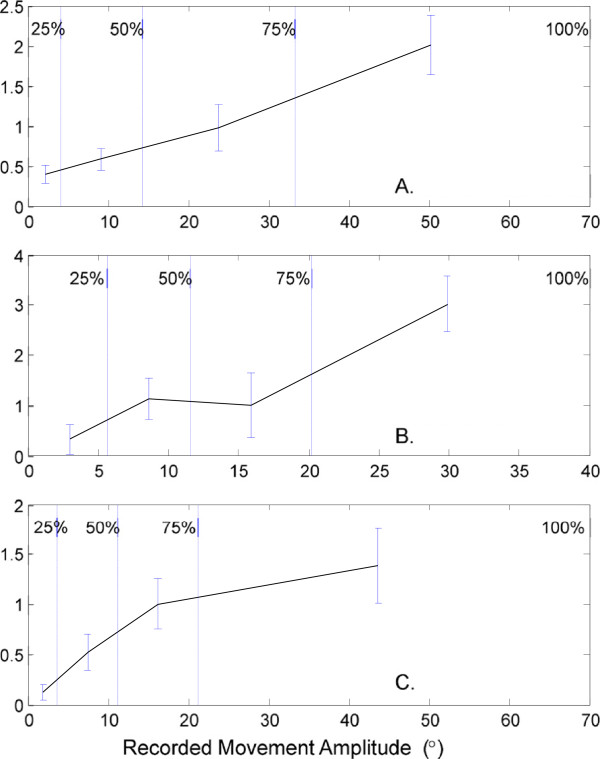
**Error improvements by the NS as a function of recorded torso movement amplitudes.** The RMSE improvement by NS was defined as RMSE_MS_ –RMSE_NS_. Recorded torso movement amplitude was defined as max – min angle. **A**: Flexion/extension, **B**: Axial rotation, and **C**: Lateral bending. Error bars indicate 95th percentile confidence intervals. 25%, 50%, 75%, and 100% denote 25^th^, 50^th^, 75^th^_,_ and 100^th^ percentile torso movement amplitudes, respectively.

Estimation errors were also quantified as a function of movement amplitudes for different torso joints (Figure 6). The torso movement amplitude was defined as the angular range (=max – min angle) of a joint in a recorded movement. Error improvement was defined as the decreased RMSE when the NS was employed in the model (=RMSE_MS_ – RMSE_NS_). The error improvements were averaged for each of the incremental intervals corresponding to 0-25^th^, 25-50^th^, 50-75^th^, and 75-100^th^ percentile of torso movement amplitudes.

In general, RMSE improves as torso movement amplitude increases. For example, for the flexion/extension joint (Figure 6A), the 75-100^th^ percentile amplitude interval, which corresponds to approximately 33-67° flexion, showed that the NS reduces RMSE by 2° on average. However, in the 0-25^th^ percentile amplitude interval (0-4° flexion), the RMSE improvement is 0.4° on average. Overall, Pearson’s correlation coefficient (*R*) is 0.35 (*p* < 0.05). Similar tendencies were observed for axial rotation (Figure 6B; *R* = 0.29, *p* < 0.05) and lateral bending (Figure 6C; *R* = 0.27, *p* < 0.05).

## Discussion

We hypothesized that during visually guided reaching movements the MS and VS make different, sometimes incompatible demands for torso movements. We modeled a means by which competing demands are resolved using a negotiation framework in the differential inverse kinematics. This framework accounted for the ability of both the MS and VS to compromise on torso movements without sacrificing hand or gaze movements by virtue of MS and VS redundancy. The negotiation function allocated torso movements after apportioning null space movements in both the MS and VS.

The results indicated that the predictive power of the estimated joint angle trajectories is improved when the negotiation framework is implemented. RMSE and end-posture estimation accuracy improved with the NS compared to models based on either the MS or VS only. These results suggest that upper body movements are more accurately described with a system incorporating *both* the manual reaching and visual gaze transition components simultaneously, as opposed to considering only one component over another exclusively.The NS generates torso movement profiles similar to a weighted average of the MS and VS estimations (Figure 2). In comparison with the MS or VS, the NS estimations are closer to the recorded movements. However, estimation errors are not always smaller from the NS than from the MS or VS. In several cases, either the MS or VS estimates are closer to the recorded movements. These apparent discrepancies may result from the effective contribution of the torso, since the estimation errors decrease when torso movement amplitude increases (Figure 6). In other words, the benefit of the NS is greater for movements requiring larger torso displacement amplitudes, which underlines the necessity to reach a compromise through negotiation when torso movements must satisfy the combination of two systems demands.

Thus the results of this study *suggest* that the CNS, in planning and organizing upper body movements, weighs the demands from each movement system and allocates the shared resources, i.e., the torso and other upper body joint movements, in accordance with weighted demands. The NS seems to have allocated different amounts of torso contribution to the MS and VS depending on target location (Figure 3, 4 and 5). Different target locations are associated with significantly different hand-to-target distances. For example, targets on the left side correspond to longer hand-to-target distances than those on the right side (69 cm versus 51 cm on average respectively, for targets below eye level at 155% reach distance), since reaching movements were performed with the right hand. The longer hand-to-target distance requires torso movement toward the target to extend the hand reaching distance. Indeed torso contribution to the MS is larger than the VS for targets on the left side, and vice versa for targets on the right side. From a biomechanical perspective this contribution is a necessity well predicted by the NS.

In the same way that torso movements vary with biomechanical demands, upper body posture can be significantly influenced by the demands for visual image acquisition [23,24]. In this regard, we may assume that targets in the mid-sagittal plane at eye level impose minimal demands for head and torso movement to orient gaze, while eccentric targets away from the mid-sagittal plane and/or eye level require more contribution from the head, neck, and torso. The decreased allocation of torso contribution to the MS (thus increased allocation to the VS) in the elevation × azimuth effects (Figures 3, 4 and 5) show consistent findings. This hypothesis is also in agreement with our previous results [18], obtained in a similar seated condition, which showed that torso axial rotation begins to appear for visual targets with azimuth angles greater than 60°.

The negotiation function may be viewed as an integration of a number of cost functions contributing to body segment coordination. It may be presumed that disruption of coordination by neurological disorders such as stroke or Parkinson’s disease affecting CNS functions may be associated with a disruption of the hypothesized negotiation process. Hence, the proposed model may find application in the analysis of neurological disorders.

In comparison with other differential inverse kinematics models of upper body movement [10,11], which have ignored visual constraints, the novelty of this study resides in a biomechanical system model allocating resources to visual information acquisition. The limitation of the current study includes the absence of eye movement recordings, which could have helped quantify VS behaviors more precisely. Given the fast speed of eye movements, however, it is posited that gaze lands on target before the hand or in the very early phase of hand reaching movement [7]. Furthermore, the participants were to read aloud the characters sequentially displayed on each target. This requirement effectively constrained the gaze to remain on target throughout the reaching movement. Hence it is assumed that eye orientation (eye in head) is equal to the angular offset between the head and target. This allows us to indirectly estimate gaze orientation using head and target positions. Further, the head and neck were modeled as a single link rotating about the C7/T1 joint, instead of separate links. However, with the complex musculature and coupling of cervical spine movements, neck movements are generally constrained by head movements [25,26].

It should be also noted that the proposed model assumes that the goals of both the MS and VS are pursued simultaneously at any given time throughout a reaching movement. However as the number of systems and goals the CNS needs to consider increases (for example, if walking, balance control, bimanual reach, etc. were to be additionally included), the simultaneous control of multiple systems, as outlined here, may no longer be an efficient solution. The RMSE still remaining in the NS system is potentially related to such additional goals, not only those for fulfilling visual and manual demands. Alternatively, the actions of the controlled systems may be divided and sequenced. Such a division has been suggested from the observation of multiple phases in joint angles and end-effector trajectories [2,27,28].

## Conclusions

Overall, the proposed model suggests that goal-directed human movements are performed by multiple cognitive and sensorimotor systems, each of which is in charge of different aspects of the overall movement goal. These goals are pursued using body segments either shared by both systems or dedicated uniquely to each system. Thus one of the crucial functions of the CNS is to allocate shared system resources and coordinate the functioning of multiple systems in a manner that maximally satisfies (and thereby resolves) the competing demands on shared resources. Hence coordination can be viewed as a process of negotiation.

## Abbreviations

CNS: Central nervous system; MS: Manual system; NS: Negotiated system; RMS(E): Root mean square (Error); RM: Recorded movement; VS: Visual system.

## Competing interests

The authors declare that they have no competing interests.

## Authors’ contributions

KHK carried out model development, experimentation, and data analysis. RBG carried out model development and data analysis. BJM conceived of the study and designed the experiments. All authors drafted, read and approved the final manuscript.
